# Involvement of O-GlcNAcylation in the Skeletal Muscle Physiology and Physiopathology: Focus on Muscle Metabolism

**DOI:** 10.3389/fendo.2018.00578

**Published:** 2018-10-16

**Authors:** Matthias Lambert, Bruno Bastide, Caroline Cieniewski-Bernard

**Affiliations:** Equipe Activité Physique, Muscle, Santé, Unité de Recherche Pluridisciplinaire Sport, Santé, Société (EA7369-URePSSS), Faculté des Sciences et Technologies, Université de Lille, Lille, France

**Keywords:** O-GlcNAcylation, slow-twitch muscle, fast-twitch muscle, glucose metabolism, exercise, skeletal muscle atrophy

## Abstract

Skeletal muscle represents around 40% of whole body mass. The principal function of skeletal muscle is the conversion of chemical energy toward mechanic energy to ensure the development of force, provide movement and locomotion, and maintain posture. This crucial energy dependence is maintained by the faculty of the skeletal muscle for being a central place as a “reservoir” of amino acids and carbohydrates in the whole body. A fundamental post-translational modification, named O-GlcNAcylation, depends, *inter alia*, on these nutrients; it consists to the transfer or the removal of a unique monosaccharide (N-acetyl-D-glucosamine) to a serine or threonine hydroxyl group of nucleocytoplasmic and mitochondrial proteins in a dynamic process by the O-GlcNAc Transferase (OGT) and the O-GlcNAcase (OGA), respectively. O-GlcNAcylation has been shown to be strongly involved in crucial intracellular mechanisms through the modulation of signaling pathways, gene expression, or cytoskeletal functions in various organs and tissues, such as the brain, liver, kidney or pancreas, and linked to the etiology of associated diseases. In recent years, several studies were also focused on the role of O-GlcNAcylation in the physiology and the physiopathology of skeletal muscle. These studies were mostly interested in O-GlcNAcylation during muscle exercise or muscle-wasting conditions. Major findings pointed out a different “O-GlcNAc signature” depending on muscle type metabolism at resting, wasting and exercise conditions, as well as depending on acute or long-term exhausting exercise protocol. First insights showed some differential OGT/OGA expression and/or activity associated with some differential stress cellular responses through Reactive Oxygen Species and/or Heat-Shock Proteins. Robust data displayed that these O-GlcNAc changes could lead to (i) a differential modulation of the carbohydrates metabolism, since the majority of enzymes are known to be O-GlcNAcylated, and to (ii) a differential modulation of the protein synthesis/degradation balance since O-GlcNAcylation regulates some key signaling pathways such as Akt/GSK3β, Akt/mTOR, Myogenin/Atrogin-1, Myogenin/Mef2D, Mrf4 and PGC-1α in the skeletal muscle. Finally, such involvement of O-GlcNAcylation in some metabolic processes of the skeletal muscle might be linked to some associated diseases such as type 2 diabetes or neuromuscular diseases showing a critical increase of the global O-GlcNAcylation level.

## Introduction

Just over thirty years ago, the O-linked N-acetyl-β-D-glucosaminylation, termed O-GlcNAcylation, was discovered inside the mouse lymphocyte cells by Torres and Hart (1). From this discovery, about 1,400 studies were focused on this field among hundreds of other known post-translational modifications. Nowadays, scientific community shows a growing interest since half of these previous studies was published in the last 5 years, and provides more and more relevant data to better characterize the impact of O-GlcNAcylation on cellular processes. It is ubiquitous from virus to plantae and metazoan, and to date around 4000 O-GlcNAc-modified proteins have been identified (2). O-GlcNAcylation seems to be an important molecular process in biology, especially since ubiquitous *OGT* and *OGA* knockout mice experiments revealed that O-GlcNAcylation balance is crucial for embryonic stem cell viability and embryonic development (3, 4); recent data also supported the essential role of O-GlcNAcylation in adult life since inducible global knockout of *OGT* dramatically increased mice mortality (5).

O-GlcNAcylation is an atypical, reversible and dynamic glycosylation. Unlike the N-and O-glycans, the O-GlcNAcylation consists of the transfer of a unique monosaccharide which is not elongated, the N-acetyl-D-glucosamine, on a plethora of nucleocytoplasmic (6) and mitochondrial proteins (7). The O-GlcNAc modification is mediated by a couple of antagonist enzymes; the OGT (uridine diphospho-N-acetylglucosamine: peptide beta-N-acetyl-glucosaminyl-transferase) transfers the monosaccharide from the UDP-GlcNAc donor to a serine or threonine hydroxyl group of a protein through a beta linkage (8), while OGA (beta-N-acetylglucosaminidase) hydrolyses the O-GlcNAc moieties from O-GlcNAcylated proteins (9). Very recent data have shown that the moiety can also be added to proteins intended for the extracellular compartment, through a distinct and structurally unrelated OGT (called EGF-OGT) which works in an OGT-independent manner (10–12). Besides its reversibility, O-GlcNAcylation is also highly dynamic. Indeed, the GlcNAc moieties can be added and removed several times along the protein lifetime, and the turn-over is shorter than the protein backbone's one. Moreover, this O-GlcNAc dynamic process could reply to many environmental conditions and physiological signals such as nutriment availability, especially from its UDP-GlcNAc donor, the last product of the Hexosamine Biosynthesis Pathway (13, 14). Finally, O-GlcNAcylation can also interplay with certain other post-translational modifications such as phosphorylation and ubiquitination [for review, see (15–17)].

Nowadays, there is evidence that some fundamental protein functions are modulated through the O-GlcNAcylation, including protein-protein interactions (18, 19), protein stability (20, 21), protein activity (22), or protein localization (23). Akin to phosphorylation, the O-GlcNAcylation is involved in almost all if not all intracellular processes (15, 24, 25) and different data demonstrated that its dysregulation can play a crucial role in the etiology of several diseases including type II diabetes (26, 27), cancer (28, 29), neurodegenerative disorders (30, 31), X-linked intellectual disability (32), neuromuscular (33), or cardiovascular diseases (34, 35), and linked to aging (36).

However, recent data showed that O-GlcNAcylation is also involved in different cellular processes of the skeletal muscle, and its potential role in many disorders related to skeletal muscle defects is still undervalued. This present review discusses the involvement of O-GlcNAcylation in skeletal muscle metabolism (in particular glucose metabolism), the impact of exercise on O-GlcNAcylation, and finally, the potential role of this post-translational modification in skeletal muscle in a context of disease such as type 2 diabetes and neuromuscular disorders.

## Relationship between O-GlcNAcylation and metabolism in skeletal muscle

In the human body, the skeletal muscle is an essential tissue that converts chemical energy into mechanical energy, i.e., contraction, to generate force and ensure some fundamental functions of the body such as movement production, posture control and thermoregulation (37). The skeletal muscle represents 40% of the total human body weight, contains 50 to 75% of all body proteins and accounts for 30 to 50% of whole-body protein turnover (37). It is a huge reservoir of nutrients (e.g., glycogen and amino acids), a great producer/consumer of energy and accounts for 30% of the resting metabolic rate in adult human. For instance, from basal state to fully state of contraction, the skeletal muscle can 300-fold increase its energy consumption within a few milliseconds (38). Interestingly, O-GlcNAcylation is known to be a cell nutrient sensor and the “O-GlcNAc signature” depends on the biological state of cells (39). Within the unitary contractile apparatus, named sarcomere, and more generally in the overall skeletal muscle cell, diverse proteins have been identified to be O-GlcNAcylated since 2004 (18, 40–43); the nature of these proteins is diversified including contractile, structural, cytoskeletal, metabolic, chaperones, mitochondrial proteins or proteins involved in signaling pathways. Thus, akin to phosphorylation (37, 44, 45), O-GlcNAcylation could play a significant role, still undervalued, in the skeletal muscle physiology.

### Exercise-mediated O-Glcnacylation changes in the skeletal muscle

Contraction is the main function of the skeletal muscle to provide force generation and ensure movement production and posture control from baseline to exercise conditions. Skeletal muscle is able to develop several adaptations with exercise by changes in contractile protein isoforms, protein turnover, metabolism, mitochondrial functions, intracellular signaling or transcriptional response [for review, see (46–48)]. As described, O-GlcNAcylation highly depends on metabolism through the Hexosamine Biosynthesis Pathway and the UDP-GlcNAc donor. Glucose metabolism has a key role in this process since about 2–5% of the glucose enters the HBP, while the remaining glucose goes to glycogen storage or glycolysis (13, 49). In addition, a study carried out on skeletal muscle, showed that HBP also depends on fatty acids, glutamate or nucleic acids (50). Among other processes, muscle contraction directly impacts glucose homeostasis. For example, exercise increases skeletal muscle glucose uptake through an insulin-dependent/GLUT4 transport pathway (51, 52) or through the activation of CamKII by calcium released upon muscle contraction (53). Thus, we can assume that O-GlcNAcylation could be modulated through muscle-training and induces several effects on muscle functions. However, although many studies reported the importance of exercise in the modulation of cardiac O-GlcNAcylation (54–59), to date there is a very few studies interesting in the skeletal muscle.

It's long recognized that metabolic flexibility concept occurs in skeletal muscle, meaning for example its ability to increase energy supply and provide sufficient and adequate “fuel” for muscle working (48). In this context, it has been recently shown that exercise could also modulate the global O-GlcNAcylation level in rat skeletal muscle (60–62). Two different types of exercise training were applied on rat: a single acute exercise run bout to fatigue and an exhausting 6-weeks interval training program, both on treadmill. Consecutively to the acute exercise, the global level of O-GlcNAcylation was not changed in the slow-twitch oxidative soleus nor in the fast-twitch glycolytic EDL as well (62) (Table 1). In contrast, a long-term training program on rat led to an increase of the global O-GlcNAcylation level in the total extract in soleus and EDL as well, whereas its level was not altered in the myofilament fraction (62). In this study, no alteration of the OGT and OGA expression was observed in both exercise protocols, suggesting a potential regulation at the activity level following the long-term exercise. However, in another study, the same authors mentioned a decreased OGA expression in the total extract of the soleus, and not in the myofilament fraction as well as in both extracts in the EDL after a similar long-term training program on rat, suggesting a differential OGA turnover which might explain, partly at least, changes in the O-GlcNAc rate between EDL and soleus (61).

**Table 1:** Difference of O-GlcNAcylation process and exercise effect on slow-twitch and fast-twitch muscles. The major muscle properties of both muscle types are also indicated.

	**Slow-twitch muscle**	**Fast-twitch muscle**
Muscle properties	Red muscle, slow contraction, oxidative metabolism, fatigue resistance *Example:* soleus	White muscle, fast contraction, glycolytic metabolism, fatigue sensitivity *Example:* EDL, white gastrocnemius
O-GlcNAcylation level	O-GlcNAcylation level in soleus > EDL	
Expression of enzymes involved in O-GlcNAcylation process	• Expression of OGT, OGA, GFAT1, GFAT2 in soleus > EDL • OGT activity on soleus > EDL	
**VARIATION OF O-GLCNACYLATION LEVEL DURING EXERCISE**		
Chronic Exercise	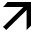	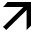
Acute exercise	Ø	Ø
Acute exercise + glutathione depletion	Ø	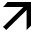

Otherwise, these O-GlcNAc adaptations following skeletal muscle activity seemed to be fully different according to the exercise protocol as well as the skeletal muscle fiber type. This could be first related to the metabolic flexibility and glucose utilization known to be different and differentially regulated depending on exercise protocols and muscle fiber types (46, 48). Little is known so far how exercise can modulate the glucose flux in the Hexosamine Biosynthesis Pathway. A study demonstrated that hindlimb skeletal muscle UDP-HexNAc concentration increased after a single swimming protocol in *ad libitum*-fed but not in fasted rats. In parallel, muscle glycogen content decreased and the GFAT activity was not altered in these conditions (63). Thus, it would be also interesting to determine how the glucose metabolism could operate a distinct modulation of O-GlcNAcylation in skeletal muscles depending on different exercise protocols since (i) the metabolism is different from fast and slow-twitch muscle, (ii) the O-GlcNAcylation is highly regulated through glucose metabolism and (iii) the glucose uptake through GLUT4 is highly modulated in skeletal muscle during exercise (52). These future directions could bring new insights in the involvement of the O-GlcNAcylation in the modulation of the beneficial effects of exercise in skeletal muscle, as a game changer to develop new strategies that counteract some muscular disorders or metabolic disorders such as obesity or diabetes mellitus.

Moreover, the O-GlcNAc adaptations post-exercise could also be directly linked to a differential modulation of the O-GlcNAc pattern and the O-GlcNAc processing enzymes between both muscle fiber types seen at resting conditions. Indeed, the O-GlcNAcylation level is higher in the slow soleus muscle compared with the fast EDL muscle (62, 64, 65); in parallel, the expression level of OGT, OGA, GFAT1, and GFAT2 is higher in soleus than in EDL (62), as well as the activity of OGT (64) (Table 1).

Finally, it is well known that the metabolism and stress response between the fast-twitch glycolytic fibers and the slow-twitch oxidative fibers are different (46). Thus, this concept could support the differential modulation of O-GlcNAcylation inducing variable consequences on cellular functions during basal and exercise conditions. During muscle activity, reactive oxygen species (ROS) are produced in skeletal muscle (66) and the O-GlcNAcylation has been shown to be involved in the modulation of oxidative stress through different signaling pathways including KEAP1/NRF2, FOXO, NFκB, and p53 (67–69). A recent study compared the O-GlcNAc pattern and O-GlcNAc processing enzymes expression between a single Diethyl Maleate (DEM) intraperitoneal injection in order to deplete glutathione, meaning oxidative stress in rat, and a single acute exercise on treadmill (60). Interestingly, in the fast-twitch white gastrocnemius, the global level of O-GlcNAcylation increased after acute exercise as well as glutathione depletion. On the contrary, in the slow-twitch soleus, no significant variation of the global O-GlcNAc level was observed (Table 1). These data suggest that the differential oxidant/antioxidant balance between slow and fast-twitch muscle could also be at the origin of the differential modulation of the O-GlcNAcylation level observed in the two muscle types. However, this complex interrelationship between cellular redox state and O-GlcNAcylation seems not to be the only mechanism involved in the O-GlcNAc regulation during exercise since the *OGT* and *GFAT* mRNA expressions were different between acute exercise and glutathione depletion in both muscles (60).

### O-GlcNAcylation could mediate the skeletal muscle glucose metabolism

Recent studies demonstrated that energy metabolism, insulin sensitivity and exercise-induced glucose uptake depends on O-GlcNAcylation (65, 70). Indeed, the muscle specific knockout of *OGT* led to the increase of glucose uptake in skeletal muscle in basal conditions (65) as well as consequently to exercise (70). Thus, the specific inhibition of O-GlcNAcylation in skeletal muscle also led to facilitation of glucose utilization in skeletal muscle, leading to greater exercise-induced glucose disposal, involving AMPK (70). Interestingly, the enhancement of glucose uptake was correlated to an increase of glycolytic enzymes activities, suggesting that mice have greater reliance of carbohydrates for energy production (65).

**Figure 1:**
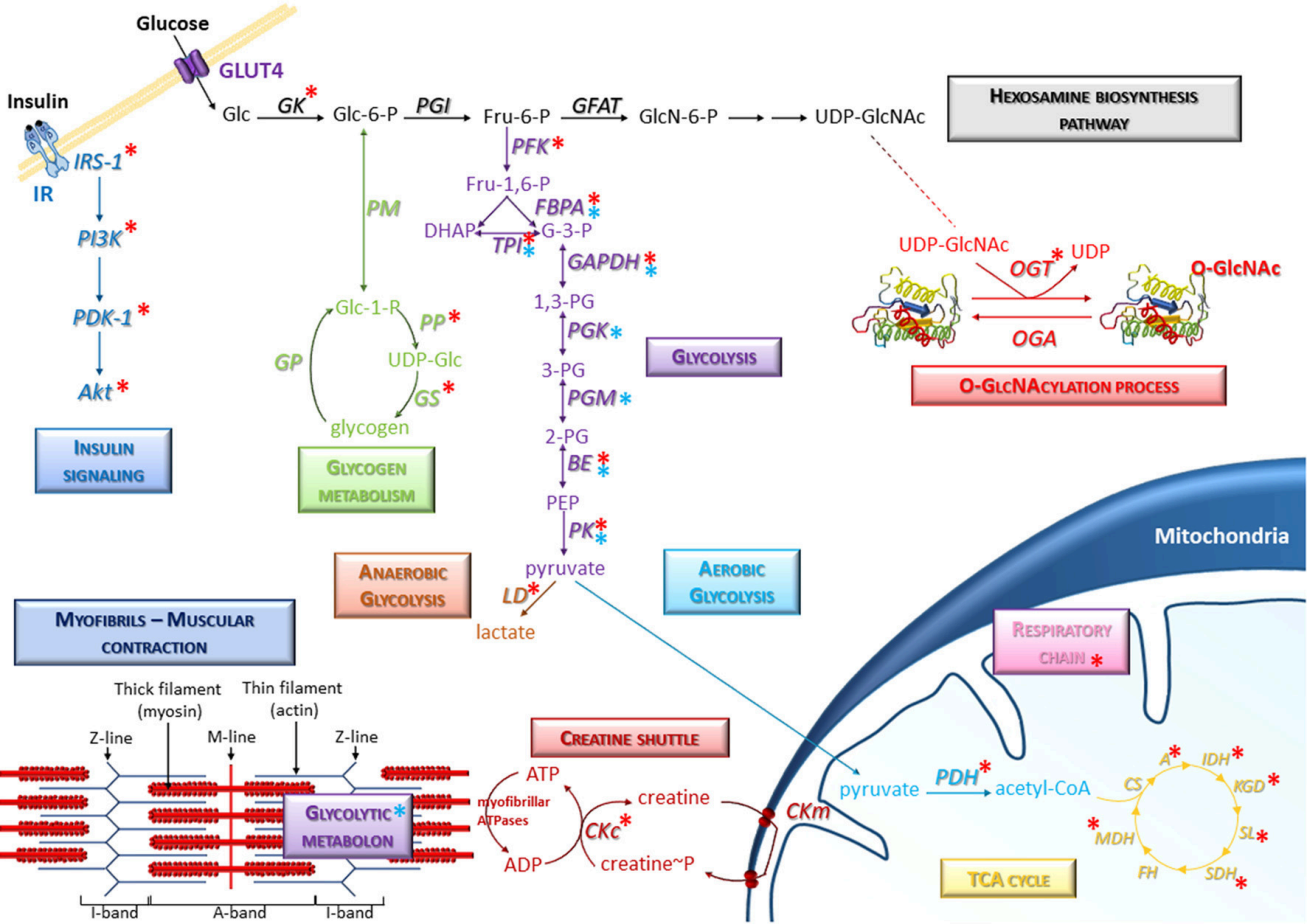
Representative scheme of the presence of O-GlcNAcylation on glucose metabolism in skeletal muscle. Several signaling and metabolic pathways are indicated, in particular the hexosamine biosynthesis pathway, glycolysis (anaerobic and aerobic glycolysis), glycogen metabolism, insulin signaling, and TCA cycle. Specific molecular components of skeletal muscle such as myofibrils and creatine shuttle are also represented. Red asterisks correspond to O-GlcNAcylated proteins; blue asterisks correspond to enzymes including in protein-protein complexes (such as the glycolytic metabolon) which could be potentially modulated consecutively to O-GlcNAcylation changes in skeletal muscle cells. A, Aconitase; BE, Beta-enolase; CKc, Creatine kinase cytoplasmic; CKm, Creatine kinase mitochondrial; CS, Citrate synthase; FBPA, Fructose-bisphosphate aldolase; FH, Fumarate hydratase; GAPDH, Glyceraldehyde-3-phosphate dehydrogenase; GFAT, Glutamine,fructose-6-phosphate aminotransferase; GK, Glucokinase; GP, Glycogen phosphorylase; GS, Glycogen synthase; IDH, Isocitrate dehydrogenase; KGD, Ketoglutarate dehydrogenase; LD, Lactate dehydrogenase; MDH, Malate dehydrogenase; OGA, O-GlcNAcase; OGT, O-GlcNAc transferase; PDH, Pyruvate Dehydrogenase; PFK, Phosphofructokinase; PGI, Phosphoglucose isomerase; PGK, Phosphoglycerate kinase; PGM, Phosphoglycerate mutase; PK, Pyruvate kinase; PM, phosphoglucomutase; PP, UDP-glucose pyrophosphorylase; SDH, Succinate Dehydrogenase; SL, Succinyl-CoA ligase; TPI, Triose-phosphate isomerase.

It is worth to note that almost all enzymes of glycolytic pathway such as phosphofructokinase (PFK), fructose bisphosphate aldolase (FBPA), triose phosphate isomerase (TPI), glyceraldehyde-3-phosphate dehydrogenase (GAPDH), beta-enolase (BE), and pyruvate kinase (PK) are O-GlcNAcylated (40, 71–76) [and for review, see (77, 78)] (Figure 1). Thus, O-GlcNAcylation may regulate the expression and/or activity of glycolytic enzymes and might consequently be involved in the regulation of glucose metabolism in skeletal muscle. To support this important role of O-GlcNAcylation as nutrient-sensor, it was first demonstrated that O-GlcNAcylation is involved in the regulation of phosphofructokinase 1 and pyruvate kinase M2 activity (71, 73). Indeed, induction of O-GlcNAcylation at serine 529 of PFK1 inhibited PFK1 oligomerization and activity, and reduced glycolytic flux as well (71). Moreover, knockdown of *OGT* led to an increased PKM2 activity (73). In this previous study, the resulting decrease of O-GlcNAc PKM2 level was associated to a decreased PKM2 expression, and to a decrease of PKM2 serine phosphorylation (73). Conversely, the increase of PKM2 O-GlcNAcylation by the use of Thiamet-G, a potent OGA inhibitor, led to upregulation of PKM2 expression and a decreased PKM2 activity (73). More recently, pharmacological inhibition of OGA and knockdown of *OGT* were associated to a respective increase and decrease of GK expression, which is the major regulator of the glucose input into cell and therefore the major regulator of glucose metabolism (79). Thus, O-GlcNAcylation is a key regulator of enzymes of glycolysis; interestingly, two downstream enzymes of PK, lactate dehydrogenase (LD) and pyruvate dehydrogenase (PDH) are also modified by O-GlcNAc moieties (74), suggesting that O-GlcNAcylation may also be an important regulator of the utilization of the glycolysis end-product, i.e., pyruvate, through the anaerobic pathway (lactate dehydrogenase) or the aerobic pathway (TCA cycle; Figure 1). Since almost all enzymes of TCA cycle are described to be O-GlcNAc modified [aconitate hydratase (A), isocitrate dehydrogenase (IDH), ketoglutarate dehydrogenase (KGD), succinyl-CoA ligase (SL), succinate dehydrogenase (SDH) and malate dehydrogenase (MDH), so as several subunits of respiratory chain complexes (40, 80, 81)], O-GlcNAcylation might play an important role in the ATP production as well (Figure 1). However, to date, neither literature mention a potential O-GlcNAcylation of citrate synthase (CS) and fumarate hydratase (FH). In the same way, the creatine shuttle, permitting the communication between ATP site consumption (i.e., myofibrillar ATPases) and mitochondria (82), could be therefore modulated by O-GlcNAcylation since creatine kinase is itself O-GlcNAcylated (40).

Many data suggest a close association between the myofibrils and the enzymes involved in the metabolism. Indeed, the fructose-bisphosphate aldolase (FBPA), enzyme of glycolysis and neo-glucogenesis, is known to be localized to the Z-line of the sarcomere in association with α-actinin within a multiprotein complex termed metabolon (83, 84). In the same way, the interaction between phosphoglucoisomerase (PGM), phosphofructokinase (PFK), glyceraldehyde-3-phosphate dehydrogenase (GAPDH), pyruvate kinase (PK) and aldolase (FBPA) also occurs with the thin filament (85, 86). These specific interactions between glycolytic enzyme complexes (termed glycolytic metabolon) and the contractile apparatus may ensure a very efficient and dynamic localized production of ATP for myosin ATPase and actomyosin interactions resulting in force development. Indeed, it was recently demonstrated that the global modulation of O-GlcNAcylation level in C2C12 skeletal muscle cells differentiated into myotubes led to the modulation of protein-protein interactions in multiprotein complexes; while this study focused on structural proteins, the proteomic data suggested that the glycolytic metabolon could be modulated by O-GlcNAcylation changes as well (Figure 1) (18). Indeed, several glycolytic enzymes (indicated by blue asterisks on Figure 1) were identified in protein-protein complexes which were modulated after the global O-GlcNAcylation changes, suggesting that the glycolytic metabolon could be potentially modulated consecutively to O-GlcNAcylation variations.

In addition, O-GlcNAcylation is also involved in the modulation of insulin pathway through the modulation of signaling proteins such as IRS-1, PI3K, PDK1, or Akt. O-GlcNAcylation of these upstream components of insulin signaling pathway occurs after the recruitment of OGT to the membrane, leading to the attenuation of insulin sensitivity (87–89) [for review, see (15, 90)]. Interestingly, the glycogen metabolism could be also modulated by O-GlcNAcylation through the regulation of glycogen synthase, O-GlcNAcylation acting as inhibitory mechanism of this enzyme (15, 91, 92); in addition, the UDP-glucose pyrophosphorylase (PP), which generates UDP-Glc, is also O-GlcNAcylated (75), suggesting that O-GlcNAcylation may be a regulator of glycogen synthesis. Thus, the O-GlcNAcylation, which depends itself of the glucose level through the Hexosamine Biosynthesis Pathway, could act as a nutritional sensor to regulate the glycolytic flow through the modification of glycolytic enzymes, the regulation of protein expression, the modulation of their phosphorylation level and/or the modulation of the metabolon.

Taken together, all these data were gained from different tissues or cell lines, and the precise role of O-GlcNAcylation on the regulation of glucose metabolism in the skeletal muscle remains to be clearly elucidated. In this context, it would also be wise to investigate the exact role of O-GlcNAcylation, not only in the regulation of enzymes expression and/or activities, but also in the modulation of these metabolon since OGT and OGA are also enriched in the Z-line and the I-band of the sarcomere (93). All together, these data strongly argue in favor of a key role of the O-GlcNAcylation process in the regulation of energy metabolism of skeletal muscle, in particular the utilization of glucose as “fuel” to provide energy to ensure muscle contraction.

## O-GlcNAcylation and skeletal muscle dysfunctions

### O-GlcNAcylation is associated to muscular atrophy

Muscle atrophy arises from a defect of the balance between protein synthesis and degradation (94, 95). Both intracellular mechanisms maintain protein homeostasis and could be potentially modulated by O-GlcNAcylation (17, 96, 97), but the role of O-GlcNAcylation in the regulation of protein synthesis and degradation is not really investigated in the skeletal muscle. However, this knowledge could be crucial since muscle atrophy is often associated with impairment of contractile and structural functions, metabolism process, and changes of phenotype fiber.

O-GlcNAcylation and skeletal muscle atrophy were firstly associated subsequently to a 14- or 28-days hindlimb unloading (HU) experiments in rat (40, 64, 93). One of the most relevant data was an opposite modulation of the global O-GlcNAc level between the slow-twitch soleus and the fast-twitch EDL (64). A decrease of the global O-GlcNAcylation level was observed in the atrophied rat soleus, while in contrast it was not altered in the non-atrophied rat EDL subsequently to the 14- and 28-days hindlimb unloading. Moreover, OGT activity was also opposite in both muscles by decreasing in soleus and increasing in EDL; however, it has been shown that OGA activity increased in both muscles (64). This first report suggested that O-GlcNAcylation could be related to the muscle atrophy and plasticity processes. Interestingly, the authors demonstrated in parallel that heat-shock proteins expression was also altered. Indeed, in the rat EDL, the HSP70 expression increased, contrary to the atrophied rat soleus after a 14-days HU (64). This heat-shock protein is known to be O-GlcNAcylated, to have lectin properties (98–100), and to have the ability to increase stress tolerance while decreasing protein degradation (101, 102). Thus, in rat EDL, the increase of global O-GlcNAc level, as well as HSP70 expression, could contribute to an improvement of stress tolerance, as suggested in cardiomyocytes (103), and prevent muscle atrophy (64, 104). Interestingly, the expression of another heat-shock protein, the alphaB-crystallin which is also known to be O-GlcNAcylated (105), was decreased in the atrophied soleus and could therefore be involved in the plasticity processes (106).

**Figure 2:**
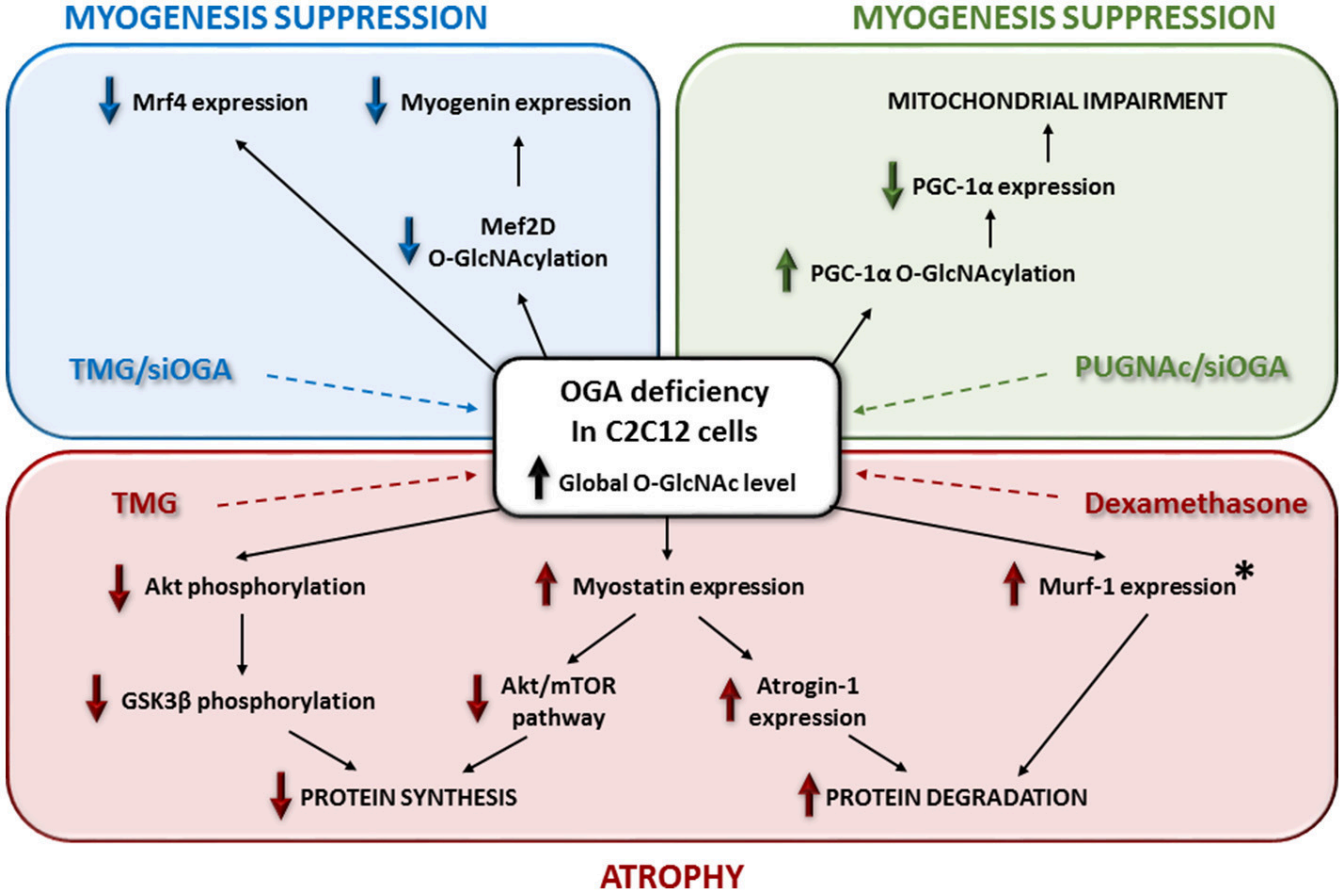
Relationships between O-GlcNAcylation, signaling pathways and muscular atrophy in skeletal muscle cells model. This figure depicts the impact of OGA deficiency on myogenesis and skeletal muscle atrophy. The asterisk means that Murf-1 overexpression occurs with the use of dexamethasone and not with the Thiamet-G in C2C12 cells.

More recently, different studies focused on the molecular pathways involved in skeletal muscle atrophy, including the relationship between O-GlcNAcylation, signaling pathways and muscular atrophy in skeletal muscle cells model (Figure 2). By using Thiamet-G, a potent inhibitor of the O-GlcNAcase, the C2C12 cells showed both a global increase of the O-GlcNAcylation and a modulation of some catabolic and anabolic pathways leading to atrophy (107). First, this study reported a significant decrease of Akt and GSK3β phosphorylation, as well as an increase of myostatin expression, which could lead to an inhibition of some anabolic pathways. Secondly, an increase of Atrogin-1 expression was reported and could lead to an improvement of some catabolic pathways (Figure 2). In this way, myostatin is a negative modulator of skeletal muscle growth and inhibits some protein synthesis pathways such as Akt/mTOR; it also promotes degradation of many sarcomeric proteins and seems to be dependent of Atrogin-1 (108). Interestingly, this report of molecular events following OGA deficiency in C2C12 could partly explain muscle wasting induction through glucocorticoid in stress conditions (109). Indeed, in C2C12 cells treated with dexamethasone, the OGA activity was decreased such as its expression (a similar molecular event was described when cells were treated with the Thiamet-G); in parallel, Murf-1 expression, leading to atrophy, increased (107) (Figure 2). It was suggested that dexamethasone could repress *OGA* gene *via* binding onto Glucocorticoid Response Element (107, 110); another mechanism could also involve OGT, known to be a cofactor of glucocorticoid receptors promoting transrepression (111). This is a relevant new insight regarding the use of glucocorticoids as the only available therapeutic treatment to maintain essential muscle functions in some muscular dystrophies (112). However, O-GlcNAc-mediated molecular mechanisms could be partly different between glucocorticoid-induced atrophy and disuse atrophy since the O-GlcNAc pattern is different, and the expression of O-GlcNAc processing enzymes is not changed (64).

In parallel, in C2C12 cells, the increase of the global O-GlcNAcylation level by the use of Thiamet-G or another OGA inhibitor such as PUGNAc or another strategy such as *OGA* knockdown, seemed suppress the myogenic differentiation of the muscle cells (113–115). Indeed, the terminal differentiation stage of C2C12 is altered through a decrease of mrf4, myogenin (113), and myoD expression (115) (Figure 2). Interestingly, a decreased O-GlcNAcylation of Mef2D, a transcriptional activator of myogenin, suppressed its recruitment to the myogenic factor promoter (114). Moreover, in the case of a global decrease of the O-GlcNAcylation level, the specific O-GlcNAc rate of PGC-1α led to its degradation and suppressed the mitochondrial biogenesis and myogenesis in C2C12 (115) (Figure 2). By the way, through OGA manipulation in C2C12 cells, O-GlcNAcylation seemed to be a negative regulator of the myogenesis. This conclusion is reinforced by the overexpression of an inactive OGA variant and the increase of O-GlcNAcylation in a rat model inducing skeletal muscle atrophy (116). In contrast, a skeletal muscle specific *OGT* knockout in mice, leading to a global decrease of O-GlcNAcylation in the tissue, did not induce muscle hypertrophy (65). Indeed, tibialis anterior, EDL and soleus of these mice showed a normal morphology and mass. Interestingly, mice exhibited reduced fat mass (65). Muscle phenotype from global *OGT* or *OGA* knockout in mice is difficult to determine since it has been shown a severe perinatal lethality, and to date there is no available data about an inducible *OGA* knockout or a skeletal muscle specific *OGA* knockout model. Along O-GlcNAcylation, all these recent studies showed complex pathways, but not fully resolved, leading to skeletal muscle atrophy.

### Skeletal muscle O-GlcNAcylation in physiopathological context

In various organs or tissues (e.g., heart, brain, pancreas, and kidney), O-GlcNAcylation was previously described to be both cell protective from an acute variation and deleterious from chronic and sustained variations especially through the impairment of glucose utilization or the glucose toxicity paradigm that may lead to the progression of several diseases [for review, see (31, 34, 36, 68, 117, 118)]. To date, one of the most defined examples is the involvement of O-GlcNAcylation in the progression of diabetes, characterized by hyperglycemia as the result of body's inability to correctly process blood glucose. Subsequently, all insulin sensitive tissues present hyper-O-GlcNAcylation and many complications. It appeared that a single nucleotide polymorphism in *MGEA5*, encoding OGA, is associated with type 2 diabetes in a mexican american population (119). Moreover, Goto-Kakizaki rats, which develop type 2 diabetes mellitus in early life, express an inactive 90 kDa isoform of OGA (120). Finally, a conditional *OGA* knockout in mice led to a low blood glucose concentration, a decreased insulin sensitivity, and to a perinatal death (121). However, the involvement of O-GlcNAcylation in the onset or in the progression of diabetes is still in debate (118) since a pharmacological inhibition of OGA in adipocytes did not cause insulin resistance or disruption of the glucohomeostasis (122).

Interestingly, among insulin sensitive tissues, the skeletal muscles are responsible for about 75% of insulin-stimulated uptake in the whole human body. In skeletal muscle, a global increase of O-GlcNAcylation induced insulin resistance (123), whereas insulin infusion led to an increase of the HBP flux and the O-GlcNAc content (124). It has been shown that muscular overexpression of GFAT in transgenic mice led to muscle insulin resistance (125, 126). In a same way, upregulation of GFAT expression and activity was described in skeletal muscle of diabetic patients (127). Moreover, overexpression of GFAT in mice is associated to a decrease of GLUT4 translocation to the sarcolemma (128), whereas transgenic mice overexpressing GLUT4 did not show alteration of GFAT expression or activity unlike the overexpression of GLUT1 (129), although both of these mice models showed an increase of glucose uptake and O-GlcNAcylation in muscle (130). Thus, the role of HBP in insulin resistance seems to be complex and still not resolved (131). Recent data provided that TRIB3 may be a novel link between HBP and insulin resistance in skeletal muscle (132).

Experiments with mice overexpressing OGT displayed muscle insulin resistance as well as hyperleptinemia (133). Pharmacological inhibition of OGA (123) or *OGA* knockdown (116) in skeletal muscle cells also induced insulin resistance. In parallel, after induction of insulin in liver, PIP3 recruited OGT from the nucleus to the membrane and caused perturbations of insulin signaling (88). Taken together, these data suggest that O-GlcNAcylation could be a link between insulin resistance and muscle impairment, since O-GlcNAcylation is involved in skeletal muscle contractility, sarcomere structuration, myogenesis, and diabetic patients often display “diabetic myopathy” (134). A significant volume of literature suggested O-GlcNAcylation linking diabetes and cardiovascular complications [for review, see (27, 35)]. In cardiac muscle from mice developing insulin resistance, mitochondrial dysfunction and changes in contractile properties were associated to an increase of O-GlcNAcylation (58). Indeed, it has been shown a correlation between increase of O-GlcNAcylation and decrease of calcium sensitivity in the cardiac tissue (135), as well as in the skeletal muscle tissue (41, 42). Interestingly, it has been shown that adenoviral transfer of *OGA* (136) or injection of a bacterial homologue of *OGA* (137), reversed the excessive O-GlcNAc content and the cardiac contractile dysfunctions. In this study, many myofibrillar proteins exhibited changes of their O-GlcNAcylation level, but the modulation of contractile properties could be also explained by a modulation of Ca^2+^ handling (138). Indeed, within an insulin resistance context in cardiac tissue, Serca2a expression was changed (58, 136), and an alteration of O-GlcNAc levels significantly affected Ca^2+^ handling, Serca2a (139) and STM1 functions (140). This role of O-GlcNAcylation should be considered also in skeletal muscle since dysfunction of contractibility as well as the Ca^2+^ handling were also measured in skeletal muscle of rat models of diabetes (141), *via* impaired Serca and GLUT4 (142).

Interestingly, OGT and OGA are highly concentrated to the sarcomere (93). Moreover, in cardiac tissue of STZ-diabetic mice, OGA, and OGT mislocalization in the sarcomere was associated to activities alteration as well as changes in the OGA interactions with actin, tropomyosin and MLC1 (137). In parallel, OGT was also mislocalized in mitochondria, the interaction between OGT and complex IV being decreased while the OGA activity decreased (143). O-GlcNAc processing enzymes distribution in the sarcomere of skeletal muscle as well as other compartments, such as mitochondria should be also investigated to better understand the involvement of O-GlcNAcylation in diabetic context. Indeed, different data support a relocalization of O-GlcNAc in atrophy or exercise muscle activity context (61, 62, 93). Recently, in C2C12 cells, an *OGA* knockdown induced insulin resistance and a decrease of the mitochondrial biogenesis with a decreased PGC1α expression (115).

In another context, it has been shown that the global O-GlcNAc level in skeletal muscle is increased in some human neuromuscular diseases (33). Especially, compared to normal muscle fibers, the O-GlcNAc signal seemed to be relocalized from the sarcolemma to the cytoplasm and nuclei in regenerative muscle fibers of muscular dystrophies, myositis and rhabdomyolysis. A strong O-GlcNAc content signal was also displayed in vacuolated fibers in sporadic inclusion body myositis, and distal myopathies with rimmed vacuoles, as well as in neurogenic muscular dystrophy. This O-GlcNAc raise could be associated with the stress response, since HSP70 expression is increased in the cytoplasmic compartment of these neuromuscular diseases. Moreover, a mutation in the *GNE* gene is known to cause distal myopathies with rimmed vacuoles. Interestingly, UDP-GlcNAc is the substrate of the GNE enzyme, and an impairment of GNE expression/activity could altered the UDP-GlcNAc content and so the O-GlcNAcylation mediated by OGT (33).

## Conclusion and perspectives

In the past ten years, more and more studies concerned O-GlcNAcylation in the skeletal muscle physiology and physiopathology. This glycosylation is mostly related to glucose metabolism, and skeletal muscle is one of the largest consumers of glucose; in addition, numerous studies showed that skeletal muscle is essential for glucose homeostasis and insulin sensitivity. Moreover, muscle plasticity allows skeletal fibers adaptations to physiological conditions in terms of contractile but also metabolism properties. Consequently, the glucose utilization will change depending on resting, wasting or exercise, as well as the fiber type composition. Interestingly, the global O-GlcNAcylation pattern in skeletal muscle changed, depending on these different conditions and fiber types. O-GlcNAcylation may be both a cause or a consequence of the modulation of the glucose utilization, in a virtuous or deleterious cycle, since most of the enzymes from the carbohydrate metabolism are known to be O-GlcNAcylated. This concept clearly raises the O-GlcNAcylation as a potential key regulator of the skeletal muscle glucose metabolism. O-GlcNAcylation has been also shown to regulate some key signaling pathways, as well cellular stress response, involved in the maintenance of the protein synthesis/degradation balance in the skeletal muscle. From these novel insights, a new paradigm is emerging considering the O-GlcNAcylation as a key factor involved in skeletal muscle physiopathology such as atrophy or insulin resistance, and more generally in neuromuscular diseases. However, O-GlcNAc involvements as a cause or consequence of skeletal muscle impairments are currently in debate. After all, it is now clear that O-GlcNAcylation is getting many involvements in the skeletal muscle physiopathology and would be confirmed in the future by larger studies of interest.

Indeed, with the exponential development of mass-spectrometry and innovative enrichment techniques, the identification of O-GlcNAc sites which are modulated subsequently to any stimuli/condition/disease will be clearly the challenge of tomorrow. In fact, O-GlcNAcylation regulates protein activity, protein localization, protein-protein interactions, and can interplay with phosphorylation or ubiquitination. This strategy will lead to a deeper understanding of the precise mechanisms by which O-GlcNAcylation can regulate skeletal muscle metabolism. Secondly, O-GlcNAc processing enzymes behavior should be more precisely investigated, since their localizations and/or interactions, and as consequence, the pattern of O-GlcNAcylation, seemed to be changed through different stimuli in skeletal muscle fibers, especially around the myofilaments. Exercise is one of these stimuli inducing complex O-GlcNAc variations, depending on the muscle phenotype, but also the kind of exercise. Importantly, due to the enhancement of glucose utilization during exercise, the O-GlcNAcylation process in skeletal muscle could be considered as a potential target to alleviate metabolic disorders. Finally, O-GlcNAcylation should be investigated in some precise muscular dystrophies or congenital myopathies, since glucose utilization is often impaired, the sarcomere could be disorganized, the mitochondria biogenesis altered, the nuclei delocalized, or the muscle plasticity changed. It will be worth knowing if O-GlcNAcylation could contribute or alleviate neuromuscular disorders or being considered as a marker of these diseases.

## Author contributions

All authors listed have made a substantial, direct and intellectual contribution to the work, and approved it for publication.

### Conflict of interest statement

The authors declare that the research was conducted in the absence of any commercial or financial relationships that could be construed as a potential conflict of interest.
